# Portable exoskeletons for upper limb rehabilitation: A systematic review

**DOI:** 10.1002/jeo2.70416

**Published:** 2025-09-22

**Authors:** Arianna Carnevale, Guido Nicodemi, Matteo Giuseppe Pisani, Alberto Lalli, Francesco Scotto di Luzio, Pieter D'Hooghe, Loredana Zollo, Emiliano Schena, Umile Giuseppe Longo

**Affiliations:** ^1^ Fondazione Policlinico Universitario Campus Bio‐Medico Rome Italy; ^2^ Research Unit of Orthopaedic and Trauma Surgery Department of Medicine and Surgery Università Campus Bio‐Medico di Roma Rome Italy; ^3^ Research Unit of Advanced Robotics and Human‐Centred Technologies Department of Engineering Università Campus Bio‐Medico di Roma Rome Italy; ^4^ Aspetar Orthopedic and Sports Medicine Hospital Doha Qatar; ^5^ Unit of Measurements and Biomedical Instrumentation Department of Engineering Università Campus Bio‐Medico di Roma (UCBM) Rome Italy

**Keywords:** exoskeleton, portable exoskeleton, rehabilitation, shoulder, upper limb

## Abstract

**Purpose:**

This systematic review evaluates the efficacy and feasibility of portable exoskeletons for upper limb rehabilitation in patients post‐stroke and with postoperative neurological complications. It focuses on motor function, range of motion (ROM), spasticity reduction and improvements in daily living activities.

**Methods:**

Following PRISMA guidelines [12], PubMed, Cochrane, and Scopus databases were searched up to October 2024 using combinations of keywords and MeSH terms such as “exoskeleton devices” and “shoulder rehabilitation.” Included studies (January 2008–October 2023) assessed portable exoskeletons for upper limb function in patients with chronic stroke or postoperative neurological complications. Excluded were studies on non‐portable robots, animal studies, protocols, those without quantitative outcomes, and those not involving human patients. The Joanna Briggs Institute (JBI) Critical Appraisal tool, and the Risk Of Bias 2 (ROB2) tool assessed study bias.

**Results:**

Five selected studies included 70 patients. Evaluated exoskeletons included the Tenodesis‐Induced‐Grip Exoskeleton Robot (TIGER), Wilmington Robotic Exoskeleton (WREX), Hybrid Exoskeleton Upper Limb 30A (HEXO‐UR30A), and Hybrid Assistive Limb (HAL). These devices demonstrated significant improvements in motor function, ROM, spasticity reduction, and kinematic parameters. High adherence and absence of severe adverse events supported feasibility.

**Conclusion:**

Portable exoskeletons are promising tools for upper limb rehabilitation post‐stroke and after neurological surgery. They enhance motor recovery and functional outcomes. However, moderate risk of bias, small sample sizes, and limited data in orthopedic contexts—especially comparisons with conventional rehabilitation—underscore the need for further high‐quality RCTs.

**Level of Evidence:**

Level IV.

**Abbreviations**
AA: affected arm
AAN‐TIGER: Assist‐as‐needed TIGER
AOU: amount of use
BBT: Box and Block Test
CG: control group (Healthy)
CO: coordination
CS: chronic stroke
DA: dominant arm
DLJ: dimensionless jerk
EFE: elbow flexion‐extension
EXO: exoskeleton
FMA‐UE: Fugl–Meyer Assessment for Upper Extremity
FOR: fraction of reach
F/U: follow‐up
GC: gravity‐compensating
HAL: hybrid assistive limb
HEXO: exoskeletal robot
HEXO‐UR30A: Hybrid Exoskeleton Upper Limb 30A
IMU: inertial measurement unit
LDLJ: logarithmic dimensionless jerk
MAL: motor activity log
MAS: Modified Ashworth Scale/maximum angular velocity
MBI: Modified Barthel Index
MMT: manual muscle testing
N: sample size
N/A: not applicable
NDA: non‐dominant arm
O‐TIGER: original TIGER
P1: Phase 1
P2: Phase 2
PCC: prospective case control
PCS: prospective case series
PD: Parkinson's Disease
PG: Parkinson's Group
PS: post‐stroke
QOM: quality of movement
RCT: randomised controlled trial
RM: repeated measures
ROM: range of motion
SAA: shoulder abduction‐adduction
SEF: shoulder/elbow/forearm
SG: Stroke Group
Sig. Outcome: significant outcome
TA: therapy assistant
TAA: shoulder abduction‐adduction
tBBT: timed box and block test
TFE: trunk flexion‐extension
TIGER: tenodesis‐induced‐grip exoskeleton robot
TLB: trunk lateral bending
TPS: time to peak speed
TR: trunk rotation
TS: total score
UA: unaffected arm
UL: upper limb
ULH: upper‐limb hemiparesis
Vm: mean velocity
WH: wrist/hand
WMFT: Wolf Motor Function Test
WR: wrist
WREX: Wilmington Robotic Exoskeleton

## INTRODUCTION

Upper limb musculoskeletal impairments profoundly affect patients' physical function and quality of life. These conditions often lead to motor deficits, spasticity, and reduced range of motion (ROM), hindering the ability to perform activities of daily living. Traditional rehabilitation methods, while beneficial, is often limited by lower training intensity, therapist dependency, and reduced adaptability to individual patient profiles. In contrast, portable exoskeletons enable consistent and intensive training with real‐time feedback [10]. This allows for greater therapy dosage and personalisation, addressing key gaps in conventional therapy delivery [21].

Portable exoskeletons are innovative assistive devices offering a data‐driven, technology‐enhanced approach to rehabilitation in orthopaedics and neurology, particularly for patients with chronic stroke or postoperative neurological complications [13]. These wearable systems support and enhance user movements with adjustable assistance tailored to individual motor abilities [16, 22]. By enabling repetitive, task‐specific training, they aim to promote neuroplasticity and motor recovery [23]. Both active and passive exoskeletons are used in upper limb rehabilitation, targeting the shoulder, elbow, wrist, and hand [5], and interpreting user intent via sensors to improve motor function and ROM. Studies have shown their effectiveness in enhancing motor outcomes and facilitating earlier, more efficient rehabilitation. They also support early mobilisation post‐surgery, mitigating muscle weakness, joint stiffness, and mobility limitations [25], and reducing the risk of atrophy and contractures through active rehabilitation [8]. While evidence for postoperative use is still limited, exoskeletons may reduce complications like deep vein thrombosis, shorten hospital stays, and improve long‐term outcomes [30].

The aim of this systematic review is to evaluate and report the application of portable exoskeletons in the rehabilitation of upper limb function. By analysing their efficacy in enhancing motor function, predicting and improving clinical outcomes, and their potential integration into rehabilitation protocols, this review seeks to provide a comprehensive understanding of the role of portable exoskeletons in advancing patient care.

## MATERIALS AND METHODS

### Eligibility criteria

A systematic review of literature was carried out in October 2024. Full‐text articles written in English were included and encompassed both prospective and retrospective studies, as well as case‐series, case‐control studies, and randomised controlled trials. The review focused on adult populations, with participants aged over 18 years. The studies selected for inclusion were those that evaluated the efficacy and feasibility of portable active and passive exoskeletons for upper limb rehabilitation. Eligible studies needed to assess at least one of the following outcomes: motor function, measured with the Fugl‐Meyer Assessment for Upper Extremity (FMA‐UE) and with the Wolf Motor Function Test (WMFT), ROM movement quality, or activities of daily living. The exclusion criteria comprised review articles, non‐clinical studies, editorials, letters to the editor, conference commentaries, protocol studies, and articles focusing on non‐portable or fixed exoskeletons. Additionally, studies that did not include patients as participants and/or lacked quantitative outcomes were also excluded.

### Information sources

**Figure 1:**
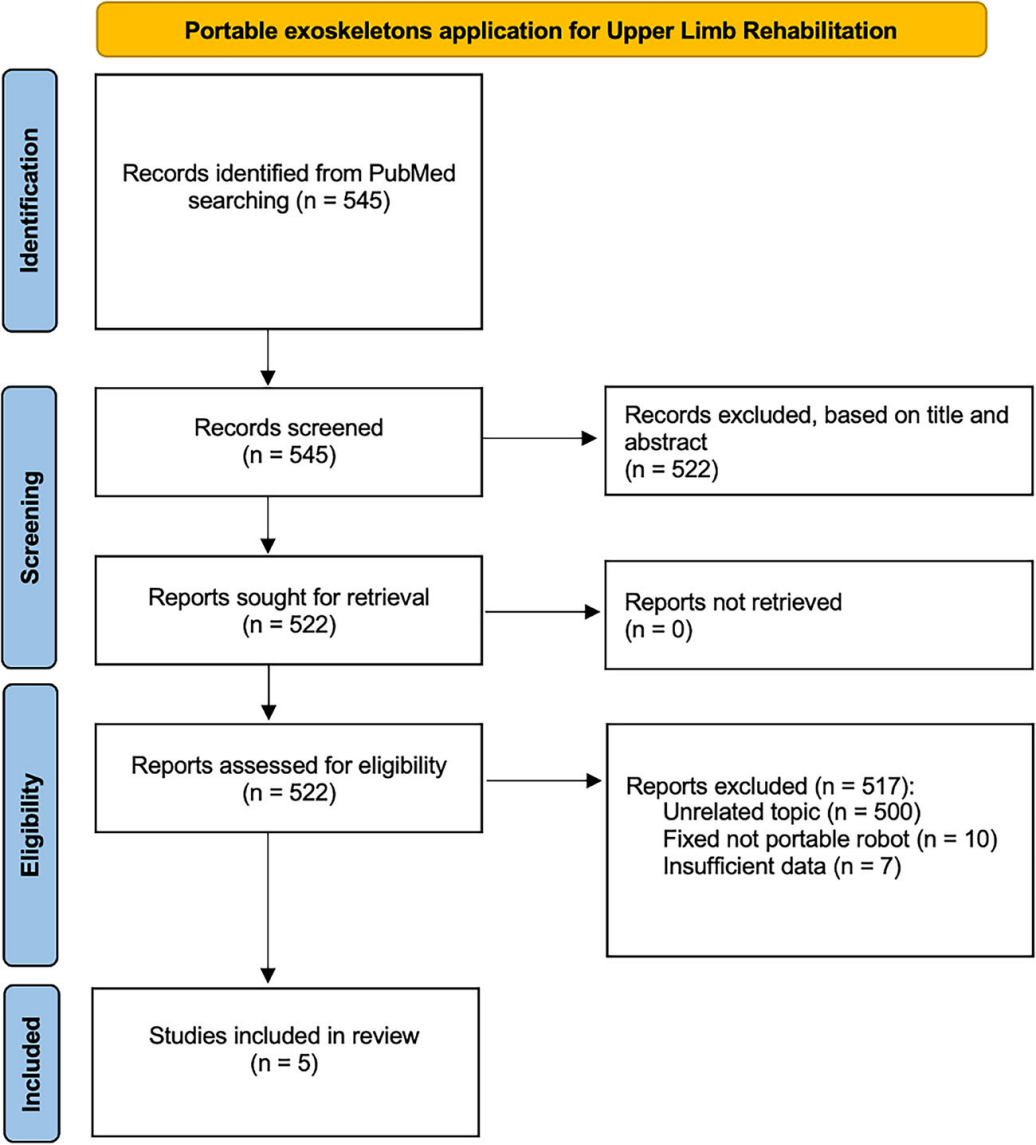
Selection flow diagram according to the PRISMA 2020 statement. [24]. For more information, visit: http://www.prisma-statement.or.

A systematic literature search was conducted using the US National Library of Medicine (PubMed/MEDLINE) database. The search retrieved a total of 545 articles from PubMed. Since the search was performed in a single database, no further deduplication across sources was necessary. This systematic review was conducted according to Preferred Reporting Items for Systematic Reviews and Meta‐Analyses (PRISMA) 2020 Guidelines (Figure 1). The main PRISMA reporting guideline primarily provides guidance for the reporting of systematic reviews evaluating the effects of interventions [12].

### Search strategy

The search strategy was designed using the Population, Intervention, and Outcome (PIO) framework. The population (P) comprised patients with upper limb movement disorders. The intervention (I) involved the adoption of portable exoskeletons for upper limb rehabilitation. The outcomes (O) included improvements in motor function, ROM, movement quality, and activities of daily living.

The detailed search syntax used in PubMed, along with time restrictions, filters, and the exact date of the search, is fully reported in Supporting Information: Appendix S1. This detailed documentation allows full reproducibility of the search process.

### Selection process

Title and abstract screening were initially performed by two independent reviewers (M.G.P., G.N.), the same reviewers performed full‐text screening of the selected articles to determine whether they met the eligibility criteria. Any discrepancies or disagreements at any stage were resolved through consultation of a third reviewer (A.L.).

### Data collection process

After selecting the eligible studies, data were extracted, including the name of the first author, year of publication, study design, level of evidence, aim, characteristics of the participants (e.g., sample size, mean age, gender distribution, etc.), pathological condition, type and model of exoskeleton and outcomes of exoskeleton application, as reported in Tables 1 and 2. For studies with incomplete or undetectable data, an initial attempt was made to contact the corresponding author for clarification. In case of non‐response or inability to provide additional data, other reviews were consulted to verify the presence of the relevant data published in other systematic reviews.

**Table 1:** Summary of study types, levels of evidence, population and aims.

First Author	Year	Study type	Level of evidence	Sample size	Condition	Mean age (SD)	Female patients %	Aim
Hsu [6]	2024	PCS	III	16	CS	56.1 ± 10.2	50.0%	To assess the effects of assist‐as‐needed TIGER on UL function in patients with CS
Iwamuro [7]	2008	PCS	III	10	ULH post‐stroke	58 ± 14	50.0%	To evaluate the effectiveness of the TA WREX, a GC orthosis, on improving reaching tasks for stroke survivors
Kim [9]	2023	RCT	I	15 (HEXO) 15 (control)	CS (ULH)	65.53 ± 8.43 (HEXO) 64.53 ± 7.72 (control)	40.0%	To evaluate the effectiveness of HEXO‐UR30A exoskeletal robot on shoulder function and ROM in CS patients
Kubota [11]	2023	PCS	III	6	Post‐op C5 palsy	65.5 ± 7.5	16.7%	To assess the feasibility and efficacy of robotic HAL shoulder exercises in patients with C5 palsy during the acute post‐op phase
Taketomi [28]	2023	PCS	III	8	CS	68.4 ± 8.38	25.0%	To evaluate the safety and effectiveness of shoulder HAL training on shoulder function and UL coordination in CS patients

Abbreviations: CS, chronic stroke; GC, gravity‐compensating; HAL, hybrid assistive limb; PCS, prospective case series; UL, upper limb.

**Table 2:** Summary of exoskeleton model, type, treated joint and output variables.

First Author	Exoskeleton model	Joint – Exoskeleton type (active/passive)	Output variables	Values
Hsu [6],	O‐TIGER, AAN‐TIGER	Wrist, hand, forearm‐active	Motor function (FMA‐UE): TS SEF WH CO	O‐TIGER: Δ = 9.2 AAN‐TIGER: Δ = 3.9 O‐TIGER: Δ = 2.6 AAN‐TIGER: Δ = 2.0 O‐TIGER: Δ = 4.3 AAN‐TIGER: Δ = 1.8 O‐TIGER: Δ = 2.41 AAN‐TIGER: Δ = 0.19
			Dexterity (BBT)	O‐TIGER: Δ = 4.0 AAN‐TIGER: Δ = 3.1
			Movement quality (MAL): AOU QOM	O‐TIGER: Δ = 0.3 AAN‐TIGER: Δ = 0.14 O‐TIGER: Δ = 0.35 AAN‐TIGER: Δ = 0.15
			Spasticity (MAS): − WR − FI	O‐TIGER: Δ = −0.21 AAN‐TIGER: Δ = −0.13 O‐TIGER: Δ = −0.21 AAN‐TIGER: Δ = −0.19
Iwamuro [7],	WREX	Shoulder, elbow‐passive	Kinematic improvements: − FOR − SPEED − TPS − Jerk	0.22 ± 0.47 −79.2 ± 67.0 cm/s −0.13 ± 0.77 −16.2 ± 66.7 cm/s³
Kim [9],	HEXO‐UR30A	Shoulder‐active	Motor function (FMA‐UE): − FMA‐UE Total − FMA‐UE Shoulder − MBI	Baseline: 19.0 ‐ F/U: 21.0 Baseline: 23.0 ‐ F/U: 23.0 Baseline: 5.0 ‐ F/U: 5.0 Baseline: 5.0 ‐ F/U: 5.0 Baseline: 64.0 ‐ F/U: 64.0 Baseline: 58.0 ‐ F/U: 59.0
			Active ROM: − Shoulder flexor − Shoulder extensor − Shoulder abductor − Shoulder adductor − Shoulder ext. rot. − Shoulder int. rot.	HEXO: Baseline: 69.0 ‐ F/U: 70.0 Control: Baseline: 68.0 ‐ F/U: 76.0 HEXO: Baseline: 23.0 ‐ F/U: 25.0 Control: Baseline: 25.0 ‐ F/U: 25.0 HEXO: Baseline: 59.0 ‐ F/U: 56.0 Control: Baseline: 55.0 ‐ F/U: 60.0 HEXO: Baseline: 27.0 ‐ F/U: 27.0 Control: Baseline: 27.0 ‐ F/U: 29.0 HEXO: Baseline: 15.0 ‐ F/U: 14.0 Contro: Baseline: 14.0 ‐ F/U: 16.0 HEXO: Baseline: 27.0 ‐ F/U: 30.0 Control: Baseline: 16.0 ‐ F/U: 16.0
			Passive ROM: − Shoulder flexor − Shoulder extensor − Shoulder abductor − Shoulder adductor − Shoulder ext. rot. − Shoulder int. rot.	HEXO: Baseline: 90.0 ‐ F/U: 97.0 Control: Baseline: 90.0 ‐ F/U: 104.0 HEXO: Baseline: 31.0 ‐ F/U: 30.0 Control: Baseline: 33.0 ‐ F/U: 33.0 HEXO: Baseline: 68.0 ‐ F/U: 67.0 Control: Baseline: 80.0 ‐ F/U: 89.0 HEXO: Baseline: 34.0 ‐ F/U: 35.0 Control: Baseline: 35.0 ‐ F/U: 35.0 HEXO: Baseline: 21.0 ‐ F/U: 21.0 Control: Baseline: 18.0 ‐ F/U: 21.0 HEXO: Baseline: 27.0 ‐ F/U: 30.0 Control: Baseline: 16.0 ‐ F/U: 16.0
			Spasticity (MAS): − Shoulder flexor − Shoulder extensor − Shoulder abductor − Shoulder adductor − Shoulder ext. rot. − Shoulder int. rot.	HEXO: Baseline: 1.0 ‐ F/U: 1.0 Control: Baseline: 2.0 ‐ F/U: 2.0 HEXO: Baseline: 1.0 ‐ F/U: 1.0 Control: Baseline: 2.0 ‐ F/U: 1.0 HEXO: Baseline: 2.0 ‐ F/U: 2.0 Control: Baseline: 2.0 ‐ F/U: 2.0 HEXO: Baseline: 1.0 ‐ F/U: 1.0 Control: Baseline: 1.0 ‐ F/U: 1.0 HEXO: Baseline: 2.0 ‐ F/U: 2.0 Control: Baseline: 2.0 ‐ F/U: 2.0 HEXO: Baseline: 1.0 ‐ F/U: 1.0 Control: Baseline: 2.0 ‐ F/U: 2.0
Kubota [11],	Shoulder HAL	Shoulder‐active	Shoulder abduction power (deltoid) MMT Shoulder abduction angle (°)	Before Shoulder HAL: 1.4 ± 0.5 After Shoulder HAL: 3.3 ± 0.5 Before Shoulder HAL: 36.4 ± 8.7 After Shoulder HAL: 140.7 ± 35.1
Taketomi [28],	Shoulder HAL	Shoulder‐active	Shoulder Joint ROM: − Voluntary ROM − Passive ROM	Voluntary ROM: Flexion Baseline: 60.0° ± 11.6° ‐ F/U: 85.6° ± 15.0° Abduction Baseline: 64.4° ± 13.2° ‐ F/U: 77.5° ± 12.5° Passive ROM: Flexion Baseline: 106.3° ± 18.7° ‐ F/U: 118.1° ± 14.9° Abduction Baseline: 93.8° ± 12.5° ‐ F/U: 105.0° ± 18.1°
			MAS score	Baseline: 9.1 ± 2.3 ‐ F/U: 5.4 ± 2.9
			Upper limb function and activity: − FMA‐UE − Maximum angular velocity	FMA‐UE: Baseline: 29.9 ± 11.1 ‐ F/U: 35.5 ± 12.1 Maximum angular velocity: Flexion baseline: 102.2 ± 41.6°/s ‐ F/U: 140.7 ± 46.8°/s Scapular plane baseline: 104.9 ± 50.5°/s ‐ F/U: 140.5 ± 45.3°/s
			Hand function tests: − ARAT − BBT	ARAT: Baseline: 11.5 ± 12.7 ‐ F/U: 16.25 ± 14.6 BBT: Baseline: 7.8 ± 14.9 ‐ F/U: 9.9 ± 14.2

Abbreviations: AAN‐TIGER, assist‐as‐needed TIGER; BBT, Box and Block Test; FMA‐UE, Fugl–Meyer Assessment for Upper Extremity; HAL, hybrid assistive limb; HEXO‐UR30A, Hybrid Exoskeleton Upper Limb 30A; MAS, Modified Ashworth Scale/Maximum Angular Velocity; MMT, Manual Muscle Testing; ROM, range of motion.

### Data items

The studies were categorised based on the type of portable exoskeleton used and the specific upper limb region targeted (e.g., shoulder, elbow, wrist and hand). Detailed information was gathered for each exoskeleton, including whether it was an active or passive system, the degrees of freedom it provided, and any unique technological features (e.g., assist‐as‐needed control, biofeedback mechanisms). Outcome measures were thoroughly documented, focusing on standardised assessment tools commonly used in rehabilitation research: (i) Motor function: FMA‐UE, WMFT; (ii) ROM: Goniometric measurements of joint angles; (iii) Spasticity: MAS scores; (iv) Movement Quality: Kinematic analyses, smoothness metrics (e.g., Jerk Score); (v) Activities of Daily Living: Functional Independence Measure (FIM), Motor Activity Log (MAL). Results were consistently collected to facilitate comparison across studies.

### Study risk of bias assessment

**Figure 2:**
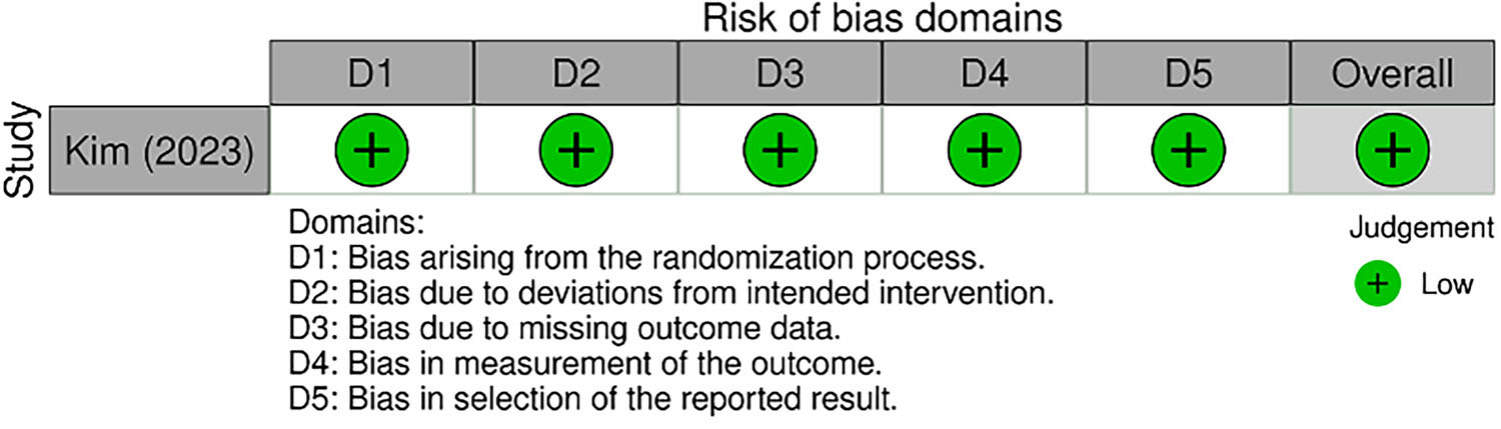
ROB2 risk‐of‐bias tool for randomised control trials.

**Figure 3:**
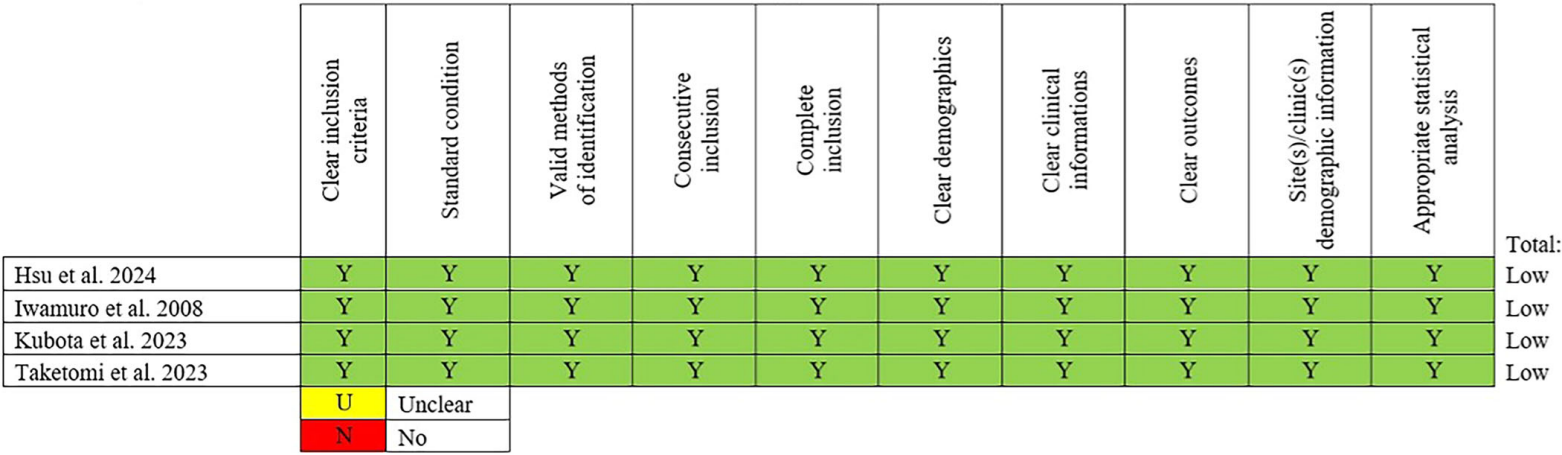
Risk of bias of included chronic strokes (CSs) evaluated using the Joanna Briggs Institute Critical Appraisal Tool for case‐series.

Two independent reviewers (G.N., M.G.P.), assessed the methodological quality of the included studies, with a third reviewer (A.L.) consulted to resolve any discrepancies that could not be settled through discussion. Two different tools were used for the methodological quality assessment: the JBI Critical Appraisal tool for case‐series studies [26], and the Risk Of Bias 2 (ROB2) tool for randomised controlled trials studies [27]. These tools are reported in Figures 2 and 3, respectively.

For case‐series, the following items were rated: clear inclusion criteria, clinical information, demographics and outcomes, standard condition, valid methods of identification and appropriate statistical analysis.

For randomised controlled trials, the ROB2 tool evaluates five specific domains: bias arising from the randomisation process, bias due to deviations from intended intervention, bias due to missing outcome data, bias in measurement of the outcome, and bias in selection of the reported result. Each domain is scored as ‘low risk,’ ‘some concerns,’ or ‘high risk’ of bias, leading to an overall risk of bias rating for each study.

Studies were rated as having a high, low, or moderate risk of bias for each component of the tool.

## RESULTS

### Study selection

A total of 545 records were found and screened for title and abstract. 522 were excluded, leaving 23 articles were selected for full‐text screening. The full‐text screening of the selected articles confirmed whether they met the inclusion criteria, resulting in the exclusion of 18 studies. The reasons for exclusion at full‐text screening included: 10 studies involved the use of fixed non‐portable exoskeleton, 7 lacked sufficient data and 1 investigated an exercise training session without the adoption of an exoskeleton device. Ultimately, a total of five articles were included in the systematic review. Following the selection of relevant studies, the data extraction process was initiated. Figure 1 displays the PRISMA flow‐chart [12].

### Study characteristics

All included studies involved the application of exoskeleton models to patients who were recruited prospectively with moderate sample sizes (ranging from 6 to 30 participants) [6, 7, 9, 11, 28]. In total, 70 patients were included in this systematic review.

Hsu et al. [6] conducted a prospective cohort study involving 16 patients with chronic stroke. The participants had a mean age of 56.1  ±  10.2 y.o., with an equal gender distribution (50% female). The study evaluated the effects of the TIGER, comparing two modes: the original TIGER (O‐TIGER) and an Assist‐as‐Needed TIGER (AAN‐TIGER). Both devices are active exoskeletons targeting the wrist, hand, and forearm. The intervention spanned nine weeks, during which participants underwent 20‐min task‐specific motor training, combined with 20‐minute robotic sessions twice per week.

Iwamuro et al. [7] presented a prospective case series with 10 stroke survivors experiencing upper‐limb hemiparesis. The participants had a mean age of 58  ±  14 y.o., and 50% were female. The study assessed the WREX, a passive, gravity‐compensating orthosis designed for the shoulder and elbow joints. The WREX facilitates reaching movements by offsetting the gravitational load on the upper limb, thereby reducing muscular effort.

Kim et al. [9] conducted a randomised controlled trial involving 30 patients with chronic stroke‐induced upper limb hemiparesis. Participants were randomly assigned to either the intervention group or the control group, each comprising 15 individuals. The mean age was 65.53  ±  8.43 years for the HEXO group and 64.53  ±  7.72 years for the control group, with 40% of the total participants being female. The intervention group received training using the HEXO‐UR30A, an active shoulder exoskeleton. The training programme lasted three weeks, consisting of ten 30‐min sessions. The control group performed standard range of motion exercises using an ergometer.

Kubota et al. [11] reported on a prospective case series involving six patients with postoperative C5 palsy. The participants had a mean age of 65.5 ± 7.5 years, and 16.7% were female. The study evaluated the feasibility and efficacy of the Shoulder HAL, an active exoskeleton targeting shoulder movements. Participants underwent an average of 11.7 HAL‐assisted shoulder training sessions during the acute postoperative phase.

Taketomi et al. [16] conducted a prospective case series with eight patients suffering from chronic stroke and severe hemiplegia. The mean age of the participants was 68.4 ± 8.38 years, with 25% being female. The study assessed the effects of Shoulder HAL training, an active exoskeleton designed to assist shoulder movements. The intervention consisted of ten sessions, each lasting 30–40 min. The exoskeleton aimed to improve motor recovery by facilitating voluntary movements of the shoulder.

### Risk of bias in studies

Two different tools were used to assess the risk of bias: the ROB2 tool, for randomised controlled trial [9], and JBI Critical Appraisal tool, for case‐series [6, 7, 11, 28]. They are reported in Figures 2 and 3, respectively.

Studies were rated as having a high, low, or moderate risk of bias for each component of the tool. All the studies were rated as having a low risk of bias concerning clear outcomes, highlighting the rigorous mathematical foundation inherent within this field.

### Results of individual studies

The detailed results of the five included studies are summarised in Table 2, which presents the type of exoskeleton, targeted joints, and outcome variables. In brief, most studies reported improvements in motor function, ROM, and spasticity, with high adherence and minimal adverse effects. Notably, Hsu et al. [6] and Taketomi et al. [28] showed substantial improvements in FMA‐UE and MAL scores, while Kubota et al. [11] reported increased muscle strength and ROM postoperatively.

## DISCUSSION

This systematic review evaluated the efficacy and feasibility of portable exoskeletons in upper limb rehabilitation. The main findings suggest that the use of portable exoskeletons leads to significant improvements in motor function, ROM, spasticity reduction, and movement quality. These outcomes are essential for enhancing patients' ability to perform activities of daily living and improving their overall quality of life [2, 19].

Portable exoskeletons effectively address the challenges of complex upper limb impairments after stroke or orthopaedic surgery by providing personalised, intensive, and task‐specific training, which is crucial for neuroplastic recovery. They overcome the limitations of traditional rehabilitation methods by offering a tailored, hands‐on approach to rehabilitation.

The studies included in this review demonstrated that both active and passive portable exoskeletons effectively enhance rehabilitation outcomes. Hsu et al. showed that the TIGER significantly improved motor function and dexterity in patients with chronic stroke [6]. The O‐TIGER outperformed the AAN‐TIGER, suggesting that consistent assistance may be more beneficial in certain patient populations. This finding aligns with previous research indicating that active engagement and repetitive practice, facilitated by exoskeletons, can promote neuroplasticity and functional recovery in stroke patients [21].

Iwamuro et al. reported that the passive Wilmington Robotic Exoskeleton (WREX) enhanced reaching tasks by compensating for gravitational forces, reducing muscular effort, and improving movement smoothness [7]. This finding supports the idea that mechanical assistance can alleviate the physical burden on patients with severe motor deficits, enabling them to perform movements they might otherwise be unable to execute [1].

In the randomised controlled trial by Kim et al. [9], the HEXO‐UR30A exoskeleton showed minimal changes in motor function compared to standard therapy. However, slight improvements in ROM were observed [14, 15]. The lack of significant differences may be attributed to the short duration of the intervention or the baseline characteristics of the participants. In contrast, Kubota et al. [11] and Taketomi et al. [28] demonstrated substantial improvements in shoulder function and spasticity reduction using the Shoulder HAL. These studies highlight the potential of exoskeletons in addressing postoperative neurological complications, such as C5 palsy, by facilitating early mobilisation and promoting motor recovery.

Overall, the evidence suggests that portable exoskeletons can effectively enhance upper limb rehabilitation by providing adjustable assistance tailored to individual needs. This personalisation is crucial, as patients exhibit varying levels of impairment and recovery potential. Exoskeletons enable repetitive, high‐intensity and task‐specific training, which are key factors in inducing neuroplastic changes and functional improvements [29].

Despite these promising findings, several limitations should be acknowledged. The included studies had small sample sizes, ranging from 6 to 30 participants, which may limit the generalisability of the results. Additionally, the heterogeneity in study designs, intervention protocols, and outcome measures presents challenges in drawing definitive conclusions. For instance, differences in the duration and intensity of interventions, as well as variations in the types of exoskeletons used (active vs. passive), may influence outcomes and complicate direct comparisons. Only one randomised controlled trial was included, with the remainder consisting of case series, which are inherently susceptible to bias. The reliance on self‐reported measures and the absence of long‐term follow‐up in some studies further limit the robustness of the evidence. Additionally, the moderate risk of bias associated with case‐series studies, prevalent due to the novel nature of the topic, may impact the strength of our conclusions.

Our literature search was confined to PubMed, Cochrane and Scopus databases, potentially omitting relevant studies from other databases, although these databases encompass most relevant articles. Furthermore, despite our initial focus on postoperative orthopaedic shoulder surgery, the paucity of relevant literature necessitated broadening our inclusion criteria to other pathologies such as stroke, which may limit the direct applicability of our findings to orthopaedic contexts.

Implications for practice suggest that while portable exoskeletons show promise, their integration into rehabilitation programmes should be approached cautiously. Clinicians should consider patient‐specific factors such as the severity of impairment, motivation levels, and accessibility to technology [3, 18]. The potential benefits of exoskeleton‐assisted therapy must be weighed against costs, the need for specialised training, and infrastructure requirements.

For policy and future research, there is a clear need for larger, high‐quality randomised controlled trials to validate the efficacy of portable exoskeletons across diverse patient populations. Studies should prioritise standardised intervention protocols and outcome measures to enhance comparability. Additionally, investigations into the long‐term effects of exoskeleton use, including sustainability of functional gains and the impact on quality of life, are essential. Research exploring the cost‐effectiveness of these devices will also inform policy decisions regarding their widespread adoption in clinical practice.

The application of exoskeletons in orthopaedics, particularly postoperative rehabilitation, remains underexplored [4]. None of the included studies directly compared rehabilitation outcomes with and without exoskeletons after orthopaedic surgeries. Future studies should address this gap by evaluating the effectiveness of exoskeleton‐assisted rehabilitation in postoperative orthopaedic patients, potentially leading to improved recovery trajectories and reduced healthcare costs. However, despite this limited scope, several of the studies included in the review presented outcomes that could be highly relevant for orthopaedic patients. Specifically, in many shoulder pathologies, axial rotation is often a key movement that is restricted, and improving this movement can significantly impact patient rehabilitation [20]. While many of the selected exoskeletons primarily assist with elevation, an important question arises: do these devices support axial rotation, a crucial degree of freedom (DoF) for shoulder function? This aspect is particularly interesting, as addressing axial rotation could have a substantial impact on improving overall shoulder mobility and function in orthopaedic patients [17]. Future studies should explore whether exoskeletons can be adapted to assist with axial rotation, thus broadening their applicability in shoulder rehabilitation.

In conclusion, this systematic review indicates that portable exoskeletons hold promise as effective and feasible tools for enhancing upper limb. They offer a solution to overcome some limitations of traditional therapy by providing personalised, intensive, and repetitive training essential for motor recovery. However, given the limitations identified, further rigorous research is needed to substantiate these findings and fully establish the role of portable exoskeletons in rehabilitation medicine.

## AUTHOR CONTRIBUTIONS

*Conceptualisation*: Umile Giuseppe Longo, Loredana Zollo, Emiliano Schena and Pieter D'Hooghe. *Methodology*: Matteo Giuseppe Pisani, Guido Nicodemi and Alberto Lalli. *Software; validation*: Umile Giuseppe Longo, Emiliano Schena and Pieter D'Hooghe. *Formal analysis*: Alberto Lalli and Arianna Carnevale. *Investigation*: Guido Nicodemi and Matteo Giuseppe Pisani. *Data curation*: Matteo Giuseppe Pisani and Guido Nicodemi. *Writing—original draft preparation*: Guido Nicodemi and Matteo Giuseppe Pisani. *Writing—review and editing*: Alberto Lalli, Arianna Carnevale, Emiliano Schena and Francesco Scotto di Luzio. *Visualisation*: Umile Giuseppe Longo, Loredana Zollo, Emiliano Schena and Pieter D'Hooghe. *Supervision*: Umile Giuseppe Longo, Loredana Zollo and Emiliano Schena. *Project administration*: Umile Giuseppe Longo, Loredana Zollo and Emiliano Schena. All authors have read and agreed to the published version of the manuscript.

## CONFLICT OF INTEREST STATEMENT

The authors declare no conflicts of interest.

## ETHICS STATEMENT

NA.

## Supporting information

Supporting information.

## Data Availability

The datasets used and/or analysed during the current study are available from the corresponding author on reasonable request.
