# Supplementary material for: Portable exoskeletons for upper limb rehabilitation: A systematic review
Source: J Exp Orthop. 2025 Sep 22;12(3):e70416. doi: 10.1002/jeo2.70416 (PMC12451468; doi:10.1002/jeo2.70416)
Supplement: Supplementary file 1 — Supporting information. [file JEO2-12-e70416-s001.docx]

Appendix

From: PubMed

From inception to October 2024

Search string:

(("Exoskeleton Devices"[MeSH] OR exoskeleton* OR "active exoskeleton" OR "passive exoskeleton" OR "wearable exoskeleton" OR "powered exoskeleton" OR "assistive exoskeleton" OR "robotic assist device" OR "robotic rehabilitation" OR "wearable robotics" OR "robotic therapy" OR "robotic orthosis" OR "rehabilitation robotics" OR "robotic system" OR "assistive device") AND  ("Shoulder Joint"[MeSH] OR shoulder* OR "shoulder surgery" OR "shoulder rehabilitation" OR "rotator cuff" OR "shoulder repair" OR "shoulder injury" OR "glenohumeral joint" OR "scapulohumeral joint" OR "shoulder arthroplasty" OR "shoulder pain" OR "shoulder disorder" OR "shoulder pathology" OR "shoulder instability" OR "shoulder dysfunction" OR "shoulder replacement" OR "rotator cuff repair" OR "shoulder prosthesis"))
